# Akt inhibitor MK-2206 promotes anti-tumor activity and cell death by modulation of AIF and Ezrin in colorectal cancer

**DOI:** 10.1186/1471-2407-14-145

**Published:** 2014-03-01

**Authors:** Ekta Agarwal, Anathbandhu Chaudhuri, Premila D Leiphrakpam, Katie L Haferbier, Michael G Brattain, Sanjib Chowdhury

**Affiliations:** 1 Eppley Cancer Center, University of Nebraska Medical Center, 985950 Nebraska Medical Center, Omaha, Nebraska 68198-5950, USA; 2 Stillman College, 3601 Stillman Blvd, Tuscaloosa, AL 35401, USA

**Keywords:** Akt inhibitor MK-2206, Ezrin T567, AIF, Cell survival, Cell death, Akt isoforms, PI3K, XIAP, Survivin

## Abstract

**Background:**

There is extensive evidence for the role of aberrant cell survival signaling mechanisms in cancer progression and metastasis. Akt is a major component of cell survival-signaling mechanisms in several types of cancer. It has been shown that activated Akt stabilizes XIAP by S87 phosphorylation leading to survivin/XIAP complex formation, caspase inhibition and cytoprotection of cancer cells. We have reported that TGFβ/PKA/PP2A-mediated tumor suppressor signaling regulates Akt phosphorylation in association with the dissociation of survivin/XIAP complexes leading to inhibition of stress-dependent induction of cell survival.

**Methods:**

IGF1R-dependent colon cancer cells (GEO and CBS) were used for the study. Effects on cell proliferation and cell death were determined in the presence of MK-2206. Xenograft studies were performed to determine the effect of MK-2206 on tumor volume. The effect on various cell death markers such as XIAP, survivin, AIF, Ezrin, pEzrin was determined by western blot analysis. Graph pad 5.0 was used for statistical analysis. P < 0.05 was considered significant.

**Results:**

We characterized the mechanisms by which a novel Akt kinase inhibitor MK-2206 induced cell death in IGF1R-dependent colorectal cancer (CRC) cells with upregulated PI3K/Akt signaling in response to IGF1R activation. MK-2206 treatment generated a significant reduction in tumor growth *in vivo* and promoted cell death through two mechanisms. This is the first report demonstrating that Akt inactivation by MK-2206 leads to induction of and mitochondria-to-nuclear localization of the Apoptosis Inducing Factor (AIF), which is involved in caspase-independent cell death. We also observed that exposure to MK-2206 dephosphorylated Ezrin at the T567 site leading to the disruption of Akt-pEzrin-XIAP cell survival signaling. Ezrin phosphorylation at this site has been associated with malignant progression in solid tumors.

**Conclusion:**

The identification of these 2 novel mechanisms leading to induction of cell death indicates MK-2206 might be a potential clinical candidate for therapeutic targeting of the subset of IGF1R-dependent cancers in CRC.

## Background

The interplay between oncogenic signal transduction pathways and their downstream mediators has been extensively characterized over the past two decades. These signaling events are transmitted by protein-protein interactions that are frequently regulated by phosphorylation events [1]. PI3K/Akt signaling is a major signal transduction cascade involved in the regulation of a number of cellular processes including cellular proliferation, survival, and metabolism. PI3K/Akt signaling has been implicated in the progression and metastasis of a wide range of cancers [2]. The Akt protein kinase, comprised of 3 isoforms (Akt1, 2 and 3), is a direct downstream effector of PI3K, which becomes fully activated by phosphorylation at the T308 and S473 sites [2,3]. Activated Akt is frequently observed in poorly differentiated tumors where it bridges the link between various oncogenic receptors and pro survival cellular functions making the tumor cells highly invasive and less responsive to chemotherapeutic drugs [2,4].

The Akt effects on aberrant cell survival are mediated by the regulation of a number of critical downstream proteins that have been implicated in apoptosis and anoikis including Bad, Caspase9, IKK, Mdm2 and FHKR [1,5,6]. Akt is also involved in cell cycle regulation by phosphorylation and inactivation of the cyclin dependent kinase inhibitors p21 and p27/kip1 [1,7]. Constitutively activated Akt has been linked to epithelial-to-mesenchymal transition (EMT) by regulating MMPs resulting in reduced cell-to-cell adhesion, increased motility and invasion. It has also been reported that Akt–driven EMT may confer the motility required for malignant progression and dissemination of cancer cells to distant organs [8,9]. Recently, we identified a new pathway by which TGFβ/PKA/PP2A signaling deactivates Akt phosphorylation leading to downregulation of IAPs, XIAP and survivin in colorectal cancer (CRC) cells [10,11].

The broad roles of this enzyme in cancer have established Akt as an attractive therapeutic candidate in cancer. Small molecule inhibitors of the PI3K/Akt pathway are being developed for clinical use. Several Akt inhibitors have been synthesized, including MK-2206, a novel allosteric kinase inhibitor of Akt [12-14]. MK-2206 binds to the pleckstrin-homology (PH) domain of Akt and thereby inhibits PDK1 binding and activation of Akt. This results in change in confirmation of Akt and its inability to localize to the plasma membrane [12-14]. MK-2206 has shown promising preclinical activity and is currently undergoing phase II clinical evaluation. Although the specific mechanisms underlying the anti-cancer activity of MK-2206 remain to be fully elucidated, MK-2206 has been shown to induce cell cycle arrest and apoptosis [12-14].

We now report that MK-2206 induces anti-tumor activity in a subset of human CRC cell lines characterized by their dependence on IGF1R signaling which leads to PI3K/Akt upregulation for cell survival [15]. Strikingly, exposure to MK-2206 resulted in the generation of 2 mechanisms of cell death, which have not previously been documented, for this drug. The MK-2206-dependent cell death of IGF1R-dependent CRC cells *in vitro* and *in vivo* was characterized by Apoptosis Inducing Factor (AIF) induction and its mitochondria-to-nuclear translocation, which is known to induce caspase-independent cell death [16,17]. Additionally, MK-2206-dependent cell death was also characterized by the inactivation of the cytoskeletal organizing protein Ezrin at T567 leading to the loss of Inhibitor of Apoptosis (IAP) family protein XIAP. It has been reported that aberrant increase of Ezrin phosphorylation at the T567 site generates increased cell survival and metastatic capabilities of cancer cells [18-21]. In summary, our results indicate that MK-2206 is a promising therapeutic candidate for treatment of IGF1R-dependent CRC characterized by PI3K/Akt signaling upregulation.

## Methods

### Cell lines and reagents

GEO [22] and CBS [23] colon carcinoma cells were cultured in serum free (SF) medium (McCoy’s 5A with pyruvate, vitamins, amino acids and antibiotics) supplemented with 10 ng/ml epidermal growth factor, 20 μg/ml insulin and 4 μg/ml transferrin at 37°C in a humidified atmosphere of 5% CO_2_[10,11] . When the cells were under GFDS (growth factor deprivation stress) [24], they were cultured in Supplemental McCoy’s (SM) medium in the absence of growth factor or serum supplements for the indicated times as described previously [24]. HCT 116 (IGF1R- independent colon cancer cell lines) [25,26] and MiaPaCa (pancreatic cancer cells with constitutive activation of IGF-1R) cells [27] were used as control to demonstrate the specificity of the dose of the kinase inhibitor. The *in vivo* experiments were carried out with GEO cells stably transfected with a GFP vector to visualize the tumor size. MK-2206 was provided by Merck and Co., Inc. MK-2206 was dissolved in DMSO for *in vitro* experiments. However, for *in vivo* experiments 30% Captisol (Cydex Pharmaceuticals) was used as a vehicle for the drug. In *in vitro* experiments, the control cells were treated with DMSO. The control animals also received 30% Captisol. AIF inhibitor, N- Phenylmaleimide was purchased from sigma.

### Proliferation assay

GEO cells were plated at a density of 8 × 10^3^ cells per well in a 96 well plate. After 72h the cells were treated with increasing concentrations of MK-2206. Cell proliferation was measured after 48h by 3-(4,5-Dimethylthiazol-2-yl)-2,5-diphenyltetrazoliumbromide (MTT) assay as described previously [15].

### DNA fragmentation assay

Cells were seeded in 96 well plates at the same density as for proliferation assays. MK-2206 was treated 72 h after plating the CRC cells. DNA fragmentation assays were performed after 48 h of treatment using a Cell Death Detection ELISA plus kit (Roche) according to the manufacturer’s protocol as described previously [24]. To confirm AIF mediated cell death, DNA fragmentation was performed by pretreating the cells with AIF inhibitor (50 μM/L) for 1 h prior to treatment with MK-2206 for 48 hrs. Additionally a DNA fragmentation assay was performed after siRNA-mediated knockdown of AIF followed by treatment with MK-2206 for 48 hrs. GEO cells were treated with XIAP siRNA for 48 h and then DNA fragmentation was performed to confirm the effect of XIAP on cell death.

### Subcellular fractionation

Cells were washed with ice-cold phosphate buffer saline (PBS) twice. The cells were suspended in 1 ml of PBS and centrifuged for 1 min at 4°C. The supernatant was removed, the pellet was dissolved in 1ml of CE buffer, and samples were vortexed for about 15 sec. The samples were kept on ice for an hour, passed through a syringe every 20 minutes and centrifuged for 1 min. The supernatant was collected and the pellet was left out to isolate nuclear extract. The supernatant was centrifuged again for 1 min to get rid of any debris. The supernatant isolated now was designated as the cytoplasmic extract and was stored at −80°C. Nuclear extract buffer was added to the pellet and the sample was vortexed for 20 seconds. The samples were kept on ice for an hour and sonicated twice for 10 seconds at 60% amplitude. The samples were centrifuged for 20 min at 4°C and supernatant collected was stored at −80°C.

### Western blot analysis and immunoprecipitation

Cells were lysed in a buffer consisting of 50 mmol/L Tris–HCl (pH 7.4), 150 mmol/L NaCl, 0.5% NP40, 50 mmol/L NaF, 1 mmol/L NaVO3, 1 mmol/L phenylmethylsulfonyl fluoride, 1 mmol/L DTT, 25 μg/mL aprotinin, 25 μg/mL trypsin inhibitor, and 25 μg/mL leupeptin. The supernatants were cleared by centrifugation at 4°C. Protein concentration was measured by bicinchoninic acid assay (Pierce) using a Biotek 96 well plate reader. Protein (30–100 μg) was fractionated by electrophoresis on a 10% acrylamide denaturing gel and transferred onto a nitrocellulose membrane (Life Science, Amersham) by electroblotting. The transfer on the nitrocellulose membrane was routinely confirmed by Ponceau S staining. The membrane was blocked with 5% nonfat dry milk in TBST [50 mmol/L Tris (pH 7.5), 150 mmol/L NaCl, 0.05% Tween 20] for 1h at room temperature or overnight at 4°C and washed in TBST. The membrane was then incubated with primary antibodies at 1:200–1:1000 in TBST overnight at 4°C. After washing with TBST for 15 min, the membrane was incubated with horseradish peroxidase–conjugated secondary antibody (Life Science, Amersham) at 1:1000 dilutions for 1h at room temperature. The proteins were detected by the enhanced chemiluminescence system (Amersham). Immunoprecipitation was performed with 500 μg of protein samples using magnetic beads (Millipore) according to manufacturer’s protocol. Antibodies were purchased from Cell Signaling for tAkt, pAkt (S473), pAkt(T308), AIF (Apoptosis Inducing Factor), pEzrin (Thr567), Akt1, Akt2, Akt3 survivin, Bad and pBad (S136). Ezrin antibody was purchased from Santa Cruz. XIAP antibody was purchased from Abcam.

### Retroviral knockdown of Akt1, Akt2 and Akt3

Small hairpin RNA sequence for Akt1si, Akt2si, Akt3si and scramblesi were cloned and expressed in a retroviral expression vector pSUPER.Retro.Puro (Oligoengine). 293T derived Phi-NX cells were used for transfection. A 19-nucleotide sequence for Akt1, Akt2 and Akt3 were designed from Dharmacon si design center. The target sequence for Akt1 5′GAGACTGACACCAGGTATT 3′ was 1634 bases while that for Akt2 5′TGAATGAGGTGTCTGTCAT 3′ selected was 301 bases downstream of 5′UTR. Akt3 target sequences selected were 5′GCAAAATGCCAGTTAATGA 3′. Another non-targeting small hairpin siRNA was used as an experimental control. The GEO cells were stably transfected with siRNA to reduce the expression of Akt1, Akt2 and Akt3. The cells were selected with Puromycin (4 μg/ml) and the resistant cells were pooled. Stable cell lines with Akt1, Akt2 and Akt3 knockdown were maintained in serum free medium with puromycin (4 μg/ml).

### RNA interference studies

XIAP siRNA (ON-TARGET plus Human XIAP (331) siRNA smart pool), AIF si RNA (ON-TARGET plus Human AIF siRNA smart pool) were purchased from Thermo scientific and transient transfections were done as per manufacturer’s protocol.

### Immunofluorescence

The translocation of AIF from mitochondria to nucleus was determined by, immunofluorescence assay. GEO cells were plated on a cover slip in a six well plate. When the cells were 60-70% confluent, culture medium with 400 nm of Mitotracker (CMX Ros, Invitrogen) was added to the cells. The cells were checked for red fluorescence under the microscope after one hour. The cells were stained, washed with growth medium and fixed by placing in ice-cold methanol for 5 minutes. The cells were washed with PBS, permeabilized by incubating with PBS containing 0.1% Triton X-100 for 15minutes and subsequently blocked with 10% normal goat serum. After one hour of blocking, the cells were incubated with primary antibody for AIF (1:100) for 2 h. Fluorescein isothiocyanate- conjugated anti rabbit antibody (FITC) was used as the secondary antibody. Nuclei were counter stained with 4′-6 diamino-2- phenylindole and mounted on glass slide in anti fade vecta shield mounting medium (vector labs). An LSM 510 microscope (Carl Ziess GmbH, Oberkochen, Germany) was used to perform laser confocal microscopy.

### Xenograft studies

All the experiments involving animals were approved by the University of Nebraska Medical Center Institutional Animal Care and Use Committee. 3–5 week old athymic nude mice (N = 16) were purchased from NCI. 7×10^6^ GEO GFP labeled cells were subcutaneously injected on one side in the right flank pad of mice and allowed to form xenografts. When the tumor size was approximately 100 mm^3^, 120 mg/kg body weight of MK-2206 was administered orally. Captisol was used as a vehicle for the drug and the control animals were treated with vehicle only. MK-2206 was given orally for 3 weeks on alternate days. The dose and the duration mentioned in the study have been provided by Merck and Co. based on standard mono therapy efficacy studies on mice. Tumor growth and body weight were measured every other day. The tumor size was measured manually with calipers, and the tumor volume was calculated using the formula (l^2^ × h × π/6). We used Near-IR enhanced Macro Imaging System Plus Cooled with the LT-99D2 with the Dual Tool dual excitation upgrade for viewing the 2D image of the tumor as well as to image the mice. All *in vivo* characterizations were confirmed in at least 3 independent control and MK-2206 treated animals.

### Terminal deoxynucleotidyl transferase-mediated dUTP nick end labeling (TUNEL) assay

The mice were euthanized after 21 days of treatment with MK-2206. The xenograft tumors were harvested after imaging to determine the size of the tumor using a microimaging system and immediately placed in 10% neutral buffered formalin fixative for 24 h. This was followed by lysate preparation and embedding in paraffin. Sections (4 μM) from paraffin embedded blocks were stained according to the Apotag terminal nucleotidyl transferase mediated nick end labeling (TUNEL) assay kit. The apoptotic rates were determined by counting the number of positively stained apoptotic bodies at 40× magnification. Fifteen different fields were randomly selected per slide for analysis. The ratio of the average number of apoptotic cells to the total number of cells counted (4000 cells each for control and treated groups) was used to determine apoptotic rates [28].

### Hematoxylin and Eosin staining and Ki67 staining

Sections (4 μM) from paraffin embedded blocks were used for H and E staining and for Ki67 IHC using antibody for Ki67 from BD biosciences. Ki67 is a non-histone nuclear antigen present in late G1, G2 and S phase of cell cycle but absent in G_0_. The dilution of Ki67 antibody used was 1:100. The proliferation rate was determined quantitatively by utilizing NIH Image J software (public domain software). Ten different, but histologically similar fields, were selected for analysis [28].

### Immunohistochemistry

The slides were deparafinized by keeping them at 60°C for 1 h and then rehydrated using graded alcohol for 5 min each. Subsequently the slides were treated with 0.3% H_2_O_2_/methanol for 10 min and then submerged in 95°C citrate buffer (pH = 7.8) for 15 min. Blocking was performed in 5% normal goat serum for 1h at room temperature and then incubated with primary antibody for tAkt (1:100) and pAkt (1:25) at 4°C overnight. The slides were treated with Biotinylated secondary antibody for 30 min at RT, followed by incubation with streptavidin peroxidase complex (Invitrogen). Reaction products were developed using diaminobenzidine containing 0.3% H_2_O_2_ as a substrate for peroxidase (Dako). Nuclei were counterstained with hematoxylin (Protocol). To determine the difference in staining intensity for total and phospho Akt, 10 different but histologically similar fields were selected per sample and the slides were analyzed using NIH image J software. The staining intensity measured by the software was plotted using Graph pad 5.0.

### Statistical analysis

Statistical analysis was performed using Graph pad 5.0 software for student’s t test. A P value of less than 0.05 was considered significant.

## Results

### Effect of MK-2206 on apoptosis of CRC cells

**Figure 1:**
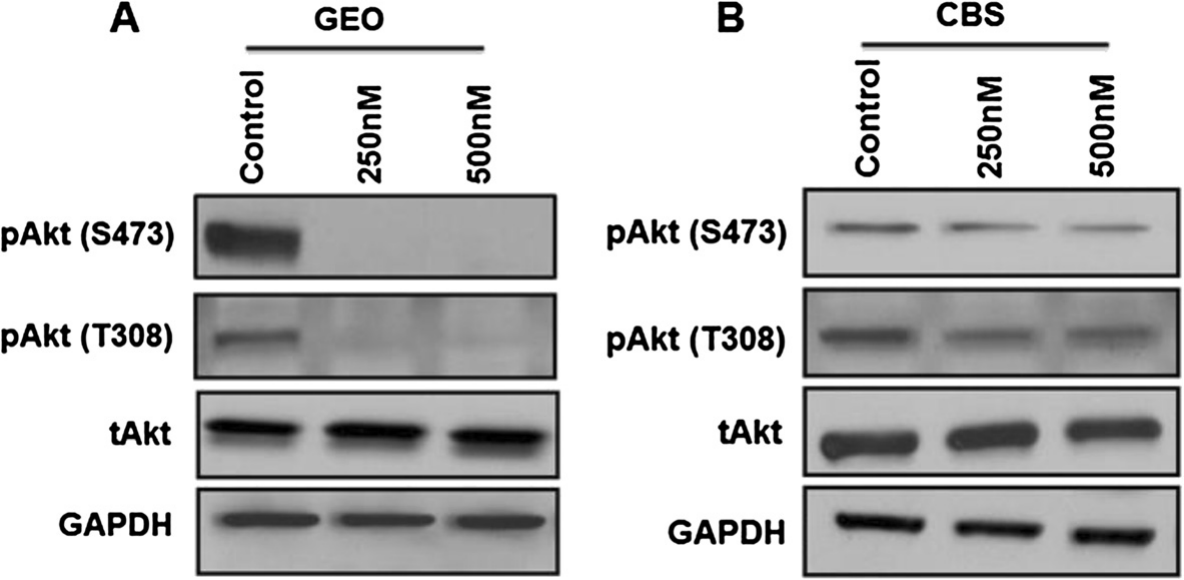
**MK-2206 inhibits Akt signaling in IGF1R-dependent CRC cells. A) & B)** Loss of pAkt at Ser473 and T308 on treatment with increasing concentration of MK-2206 for 72 hours in GEO and CBS cells respectively. GAPDH is used as a loading control.

**Figure 2:**
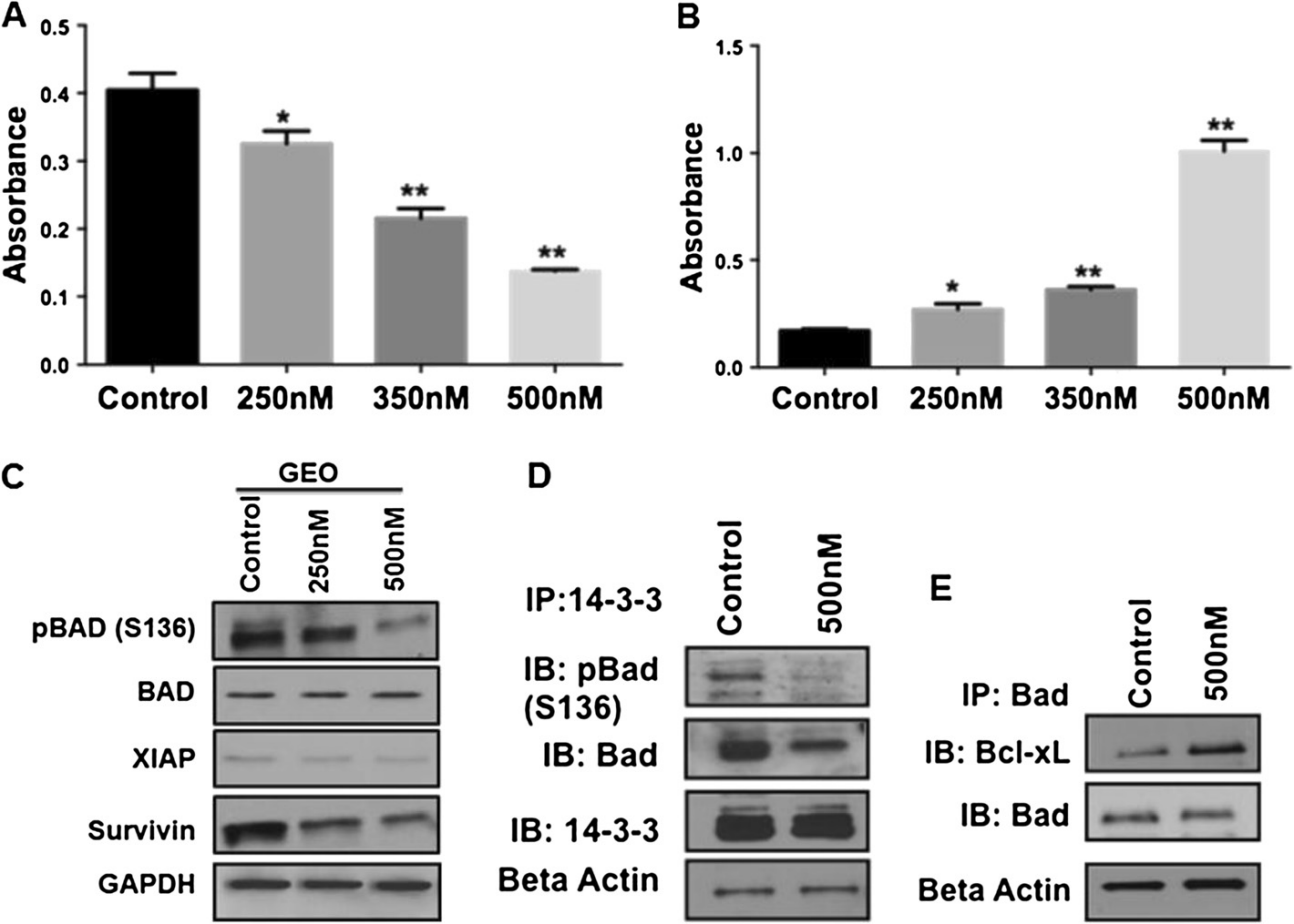
**MK-2206 affects cell proliferation and cell death *in vitro. *A)** MTT analysis shows reduction in cell proliferation on treatment with MK-2206. **B)** DNA fragmentation showing an increase in cell death with increasing concentration of MK-2206. **C)** Western blot analysis of various apoptotic members as pBad (Ser136), XIAP and Survivin. **D)** IP for 14-3-3 to determine the interaction with pBad (Ser136) and Bad showing a loss in the interaction on treatment with MK-2206. **E)** IP for Bad to determine the interaction with anti-apoptotic protein Bcl-x_L_. (* = P < 0.01 and ** = P < 0.001).

MK-2206 inhibits the phosphorylation of Akt at both Ser473 and Thr308 in two IGF1R-dependent GEO and CBS colon cancer cell lines. However the total Akt protein levels remain unchanged (Figure 1A, B). IGF1R-independent HCT116 cells [25,26] showed a marginal loss of pAkt (S473); however MiaPaCa cells with constitutive activation of IGF1R [27] showed a robust loss of pAkt with MK-2206 treatment (Additional file 1: Figure S1). We performed MTT assays to study the effect of MK-2206 on proliferation of IGF1R-dependent colon cancer cells. MK-2206 treatment for 48 h showed a concentration dependent reduction in cell proliferation (Figure 2A). The IC_50_ value of MK-2206 for GEO cells was observed to be 350 nm. Treatment with 500 nm of MK-2206 reduced cell proliferation by approximately 75%. DNA fragmentation assays were performed to determine the effect of MK-2206 treatment on cell death. It was observed that cell death increased in a concentration dependent manner on treatment with MK-2206 as shown in (Figure 2B). Treatment with 500 nm of MK-2206 increased cell death by approximately 85% as compared to control. Western blot analysis of various apoptotic markers revealed a decrease in Bad phosphorylation at Ser136 following treatment with MK-2206. (Figure 2C). Bad can undergo phosphorylation at two sites (Ser112 and Ser136). Akt preferentially phosphorylates Bad at Ser136 [29]. Phosphorylated Bad at Ser136 associates with cytoplasmic14-3-3 proteins,. Treatment with MK-2206 results in reduced interaction of pBad with 14-3-3 due to increased cell death (Figure 2D). On the other hand dephosphorylated Bad interacts with Bcl-x_L_ a pro-survival molecule, and inactivates it to generate cell death [29]. We observed that there was an increase in the interaction of Bcl-x_L_ with total Bad on treatment with MK-2206 which results in more inactivation of Bcl-x_L_ thus leading to increased cell death (Figure 2E). Furthermore, we observed a reduction in the interaction of Bad with 14-3-3 on treatment with MK-2206 (Figure 2D). It has been determined previously that there is an increase in the expression of IAPs (Survivin and XIAP) in colon, lung and breast cancer. There was an increase in cell death on transient knockdown of XIAP as determined by DNA fragmentation, which confirms that XIAP is responsible for increased survival of cells by inhibiting caspase-mediated cell death (Additional file 1: Figure S2). We observed a reduction in the expression of survivin and XIAP on treatment with MK-2206 *in vitro and in vivo* (Figure 2C*,* Additional file 1: Figure S3)*.* Therefore, MK-2206 regulates aberrant cell survival of CRC cells by down regulating IAPs in CRC cells.

### MK-2206 inhibits colon tumor xenograft growth

**Figure 3:**
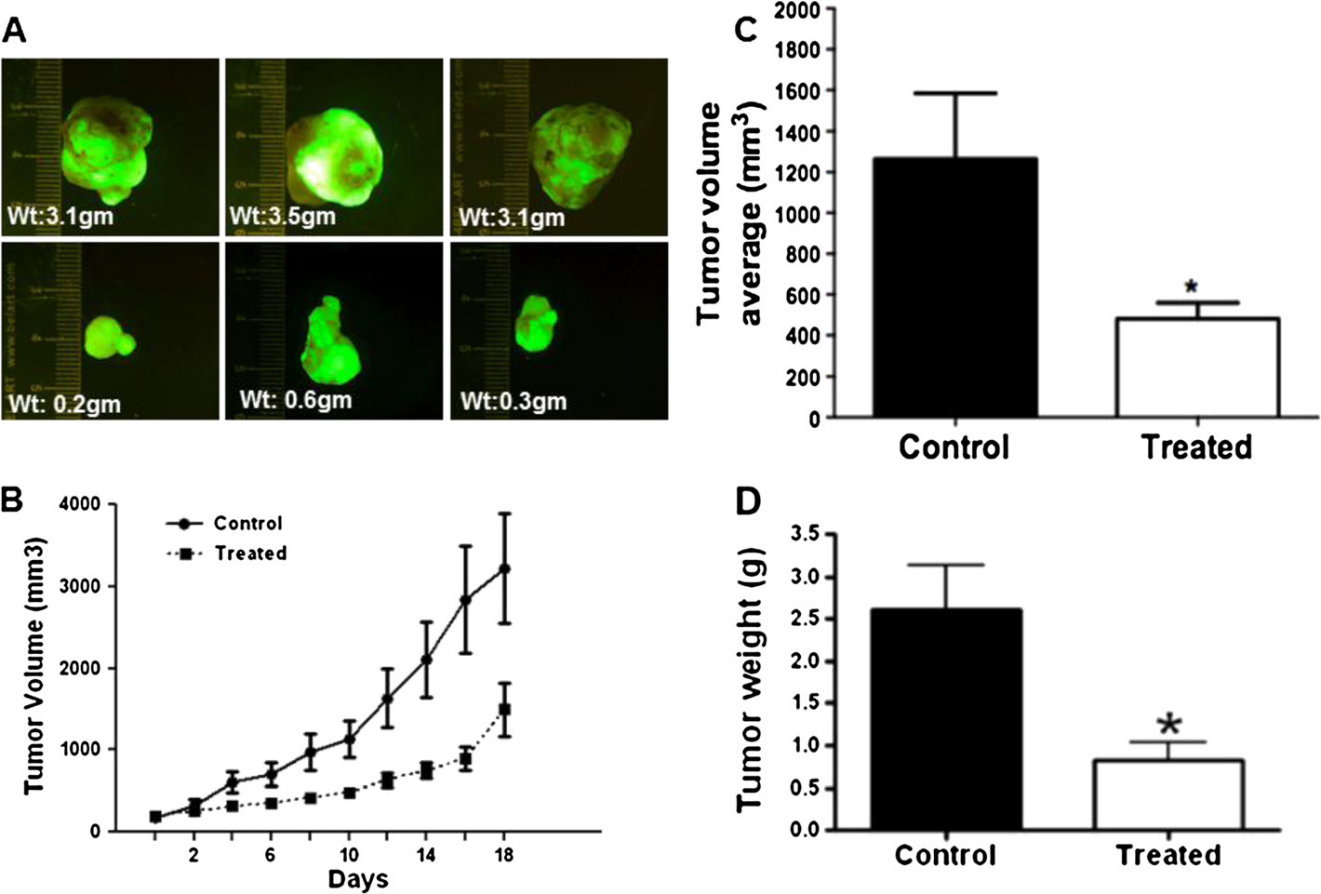
**MK-2206 inhibits the growth of colon tumor xenograft. A)** Reduction in tumor size on treatment with MK-2206. **B)** Reduction in tumor volume in treated animals as compared to control animals. **C)** Reduction in the average tumor volume in animals treated with MK-2206 as compared to control animals. **D)** Reduction in tumor weight on treatment with Akt kinase inhibitor. (* = P < 0.01 and ** = P < 0.001).

The antitumor activity of MK-2206 on GEO colon cancer xenografts was determined by injection of GEO-GFP cells subcutaneously into the flank of athymic nude mice. One week after implanting the cells, MK-2206 was administered at 120 mg/kg body weight by oral gavage for three weeks on alternate days. As shown in Figure 3A, MK-2206 significantly inhibits tumor growth. The tumor volume was found to be significantly reduced in MK-2206 treated animals (P < 0.01) as compared to control animals (Figure 3B, C). The excised tumors from control animals showed an average weight of 2.5 g compared to treated animal tumors weighing approximately 0.8 g. (Figure 3D). Importantly, there was no significant decrease in the body weight in treated animals compared to control (Additional file 1: Figure S4).

**Figure 4:**
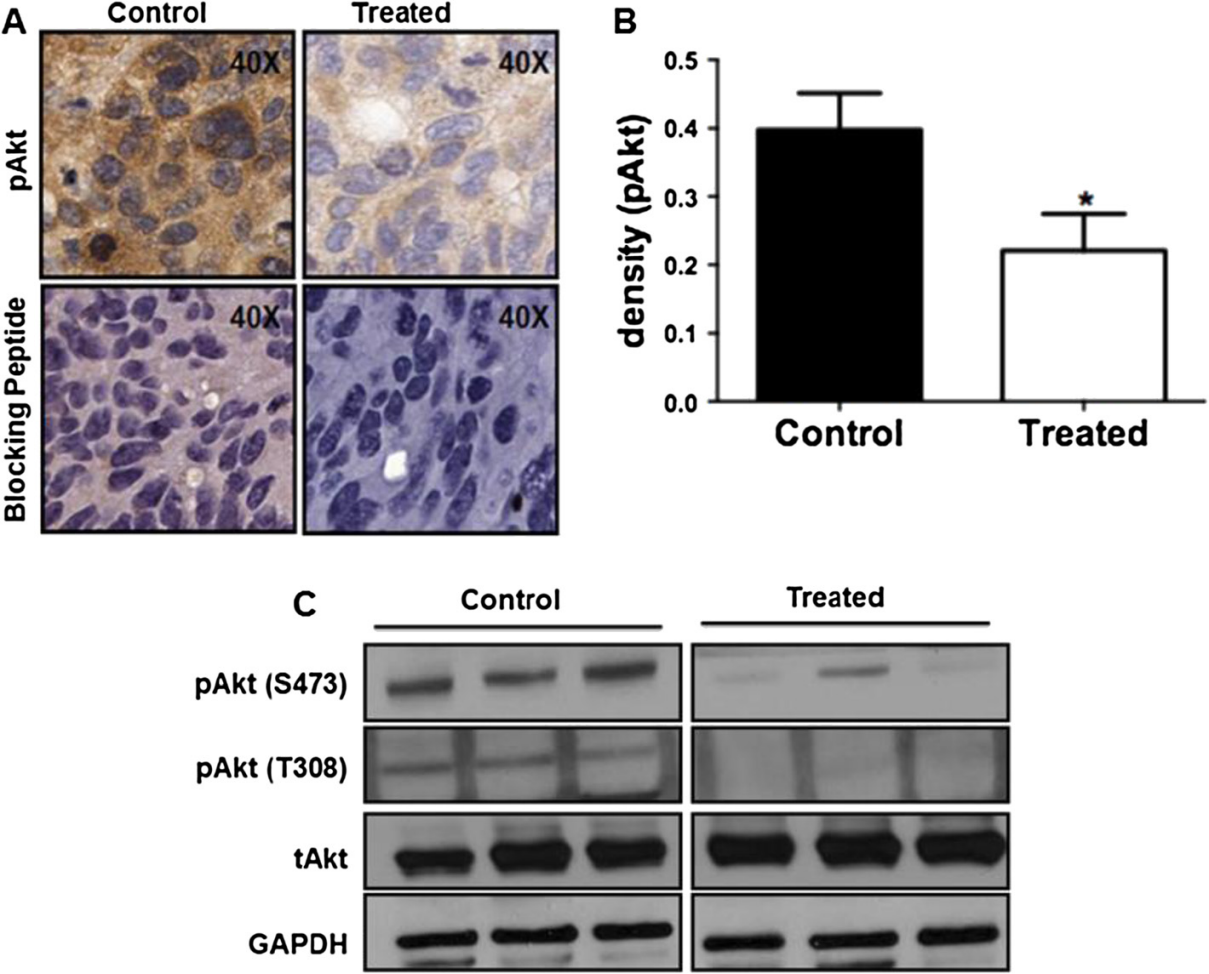
**MK-2206 inhibits Akt signaling *in vivo*: A)** IHC images showing a reduction in pAkt at Ser473. **B)** Relative quantification was performed, followed by statistical analysis to determine the decrease in phosphorylation of Akt at Ser473 on treatment with MK-2206. **C)** Western blot analysis to confirm the loss of pAkt at Ser473 and Thr 308 in treated animals. (* = P < 0.01 and ** = P < 0.001).

The expression of pAkt S473 was found to be reduced by treatment with MK-2206 *in vivo* by IHC (Figure 4A). Densitometry of the IHC images showed a significant reduction in the expression of pAkt S473 in treated animals as compared to control animals (p < 0.02) as shown in Figure 4B. The loss of phosphorylation of Akt was further confirmed by western blot analysis of MK-2206-treated tumor tissue lysates showing a reduction in pAkt at both S473 and T308 sites, in comparison to the control xenograft tumors (Figure 4C). However the change in total Akt (termed here as tAkt) was not statistically significant (Additional file 1: Figure S5, Figure S6).

### MK-2206 inhibits cell proliferation and cell death in vivo

**Figure 5:**
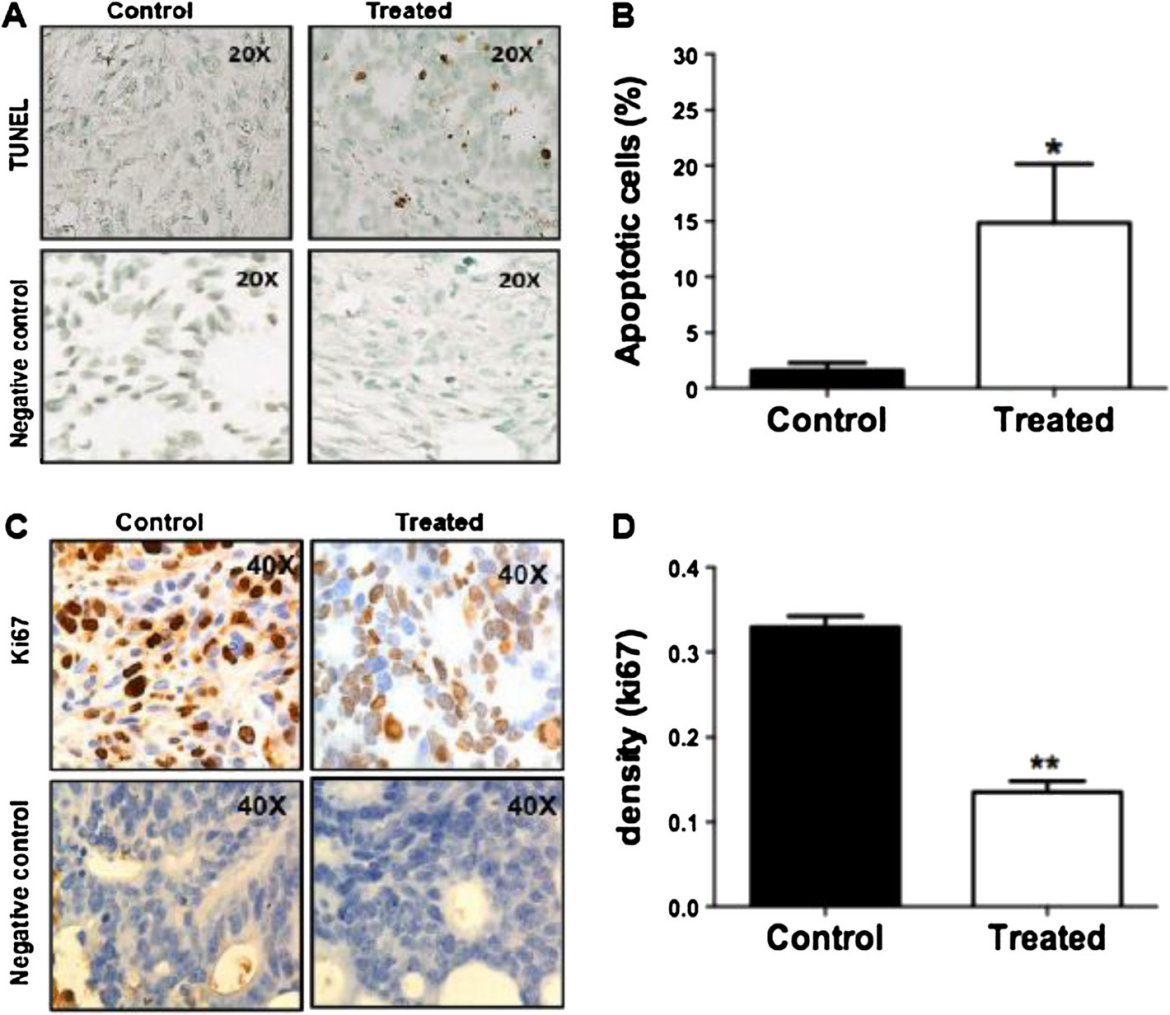
**Increased cell death and decreased cell proliferation on treatment with the allosteric Akt kinase inhibitor:** TUNEL and Ki67 IHC was performed on control and treated samples **A)** Increased cell death on treatment with the inhibitor. **B)** Relative quantification was performed followed by statistical analysis to quantify the increase in death. There was a significant increase in cell death in treated animals. **C)** Shows a loss in cell proliferation on treatment with MK-2206. **D)** Bar graphs representing a highly significant loss in Ki67 staining in treated animals.

H&E staining indicated that MK-2206 treatment induced an increase in necrosis that was observed by scanning the entire tissue section using an image scanner and visually inspecting the necrotic areas (Additional file 1: Figure S7). Cell death (quantified by TUNEL assay) was also observed to be significantly increased following MK-2206 treatment (Figure 5A, B). MK-2206 treatment also resulted in reduced cell proliferation as measured by Ki67 staining (Figure 5C, D). Additional file 1: Figure S8 shows the images of control and treated mice prior to euthanization.

### Mechanisms of cell death by MK-2206

**Figure 6:**
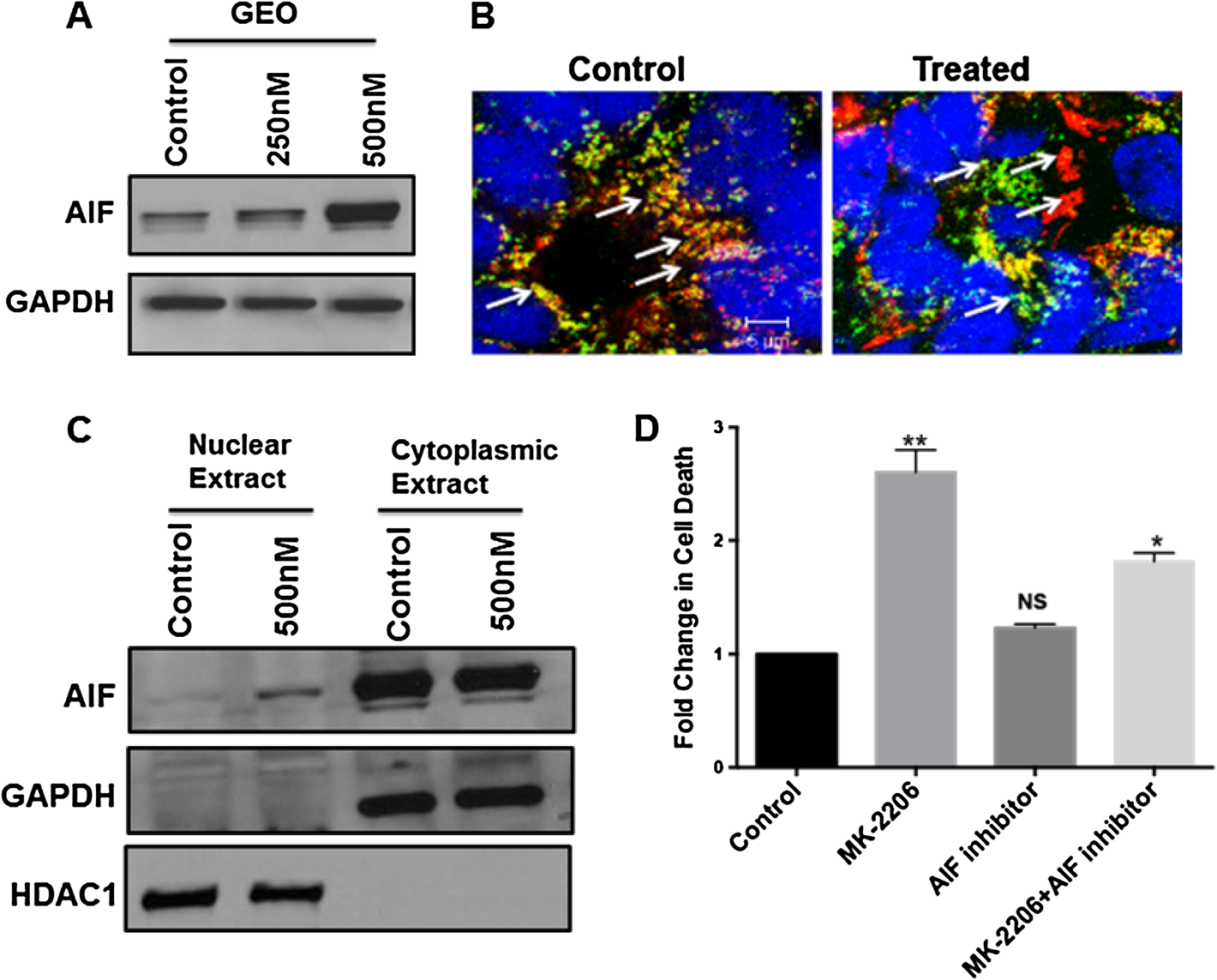
**Increase in the expression and translocation of AIF on treatment with MK-2206 mediates cell death: A)** western blot analysis showing an increase in the expression of AIF on treatment with MK-2206. Immunofluorescence was performed to study the translocation of AIF from mitochondria to the nucleus during cell death. **B)** Confocal images showing a reduced co-localization of mitotracker (red) and AIF (green) in treated as compared to control cells. **C)** Cellular fractionation to separate nucleus from cytosol was performed followed by western blot analysis for AIF. HDAC1 and GAPDH were used as compartmentalization control for nucleus and cytosol respectively. **D)** DNA fragmentation after treatment with AIF inhibitor results in reduction in cell death in presence and absence of MK-2206 thus confirming that MK-2206 causes AIF mediated cell death.

MK-2206 treatment promotes cell death both *in vitro* and *in vivo*. We characterized the molecular effects underlying MK-2206 mediated cell death in colon cancer cells. Western blot analysis showed that there was an increase in the expression of AIF protein after treatment with MK-2206 (Figure 6A). The mechanism by which the loss of pAkt might be related to this induction is not known. Cregan et al. [30] previously reported that AIF is responsible for caspase-independent apoptosis [30] by undergoing translocation from the mitochondria to nucleus. To determine the migration of AIF, we prepared nuclear and cytoplasmic extracts of untreated cells and cells treated with MK-2206 at 500 nm (since there was a higher increase in expression of AIF at 500 nm). Immunoblot analysis indicated higher AIF expression in nuclear extracts of cells treated with MK-2206 as compared to nuclear extracts of untreated cells (Figure 6C), thus confirming that treatment by MK-2206 stimulates translocation of AIF to the nucleus. Translocation of AIF was further confirmed by immunofluorescence using confocal microscopy (Figure 6B). AIF mediated cell death was further confirmed by AIF inhibitor N Phenylmaleimide [16,17]. Treatment with the AIF inhibitor at a concentration of 50 μM/L for 1h prior to treatment with MK-2206 for 48 h shows a reduction in cell death thus confirming MK-2206 mediated cell death is through stimulation of AIF (Figure 6D). Additionally loss of AIF by siRNA mediated knock down results in reduction in cell death in presence of MK-2206 as determined by DNA fragmentation assay (Additional file 1: Figure S9).

**Figure 7:**
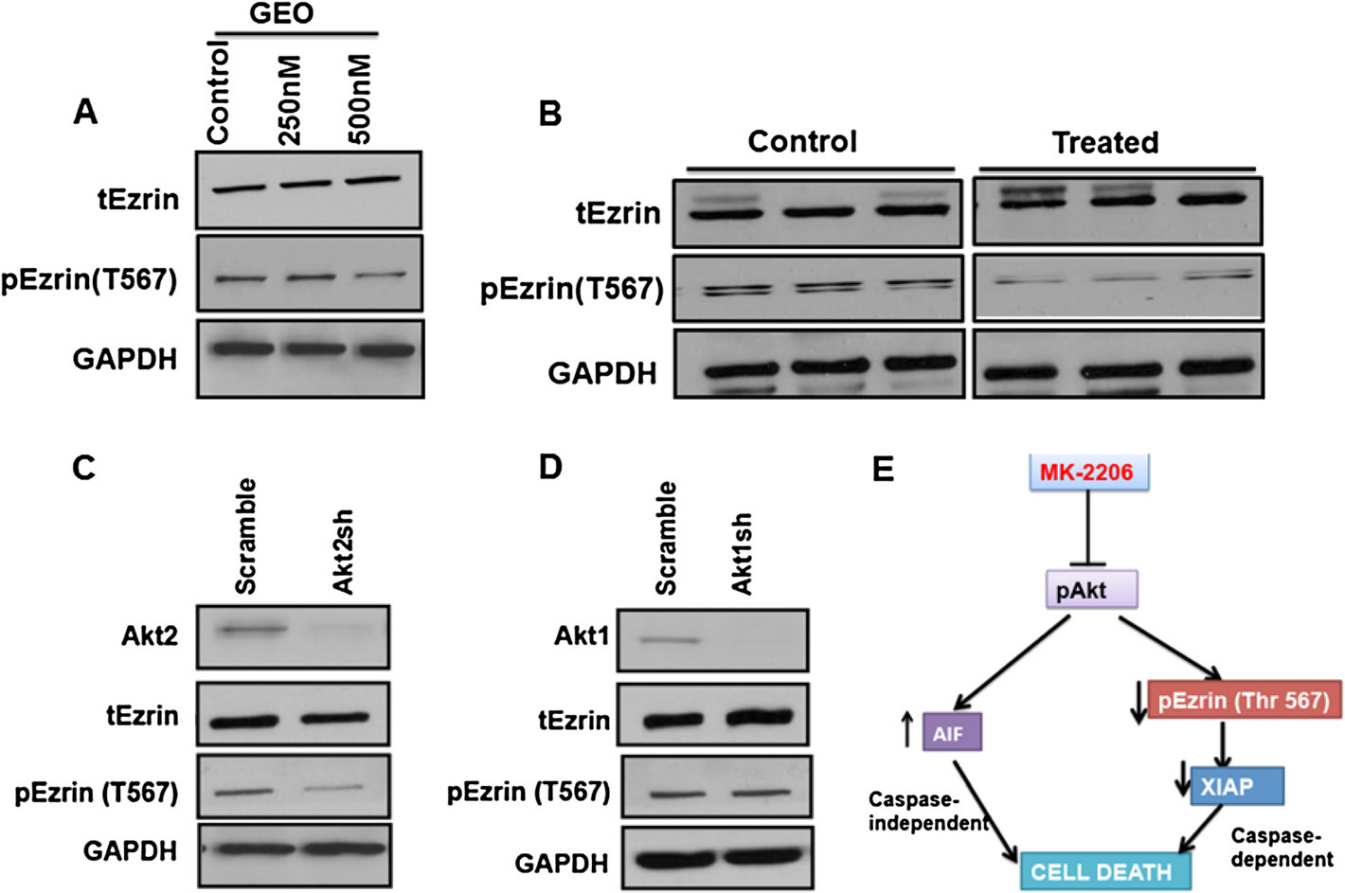
**Loss of pEzrin on treatment with MK-2206 mediates cell death: A)** Western blot analysis showing a reduction in the expression pEzrin (T567) on treatment with MK-2206 *in vitro*. **B)** Treatment with MK-2206 reduces the expression of pEzrin (T567) *in vivo.* Stable knockdown of Akt2 was performed in GEO cells. **C)** Western blot analysis showing a loss of pEzrin (T567) on knockdown of Akt2. No change in total Ezrin was observed on loss of Akt2. **D)** Western blot analysis showing loss of Akt1 does not affects the expression of pEzrin (T567). GAPDH is used as a loading control. **E)** Overall mechanism for induction of cell death by MK-2206. Akt kinase inhibitor MK-2206 mediates cell death by two different mechanisms. Loss of phosphorylation of Akt results in induction and translocation of AIF from the mitochondria to the nucleus, where it results in DNA fragmentation. On the other hand treatment with MK-2206 results in loss of pEzrin (T567), which results in loss of XIAP thus mediating cell death.

In addition to caspase-independent cell death, we also observed caspase-dependent cell death through XIAP downregulation following treatment with MK-2206 (Figure 2D). It has been shown that Akt2 regulates phosphorylation of Ezrin at T567 leading to the translocation and activation of the Na^+^–H^+^ exchanger (NHE3) [31] and NHE regulatory factor 1 (NHERF1) supports Akt dependent cell survival [21]. We observed that MK-2206 might inactivate Ezrin by affecting its phosphorylation at the T567 site (Figure 7A, B) *in vitro* as well as *in vivo*. The loss of Ezrin phosphorylation is known to affect cellular survival and proliferation [21]. Stable retroviral knockdown of Akt2 also results in reduction in Ezrin phosphorylation at T567. However there was no change in expression of total Ezrin on knockdown of Akt2 as shown in (Figure 7C). Interestingly no such loss of phospho Ezrin T567 was observed with Akt1 and Akt3 knockdown (Figure 7D, Additional file 1: Figure S10). Furthermore, Ezrin knock down resulted in complete loss of XIAP and survivin (Additional file 1: Figure S11). Therefore, it appears that Akt2 plays an important role in regulating cell survival mediated by the Akt2-pEzrinT567-XIAP axis. MK-2206 treatment caused AIF activation and Ezrin dephosphorylation at the T567 site and, ultimately, this leads to loss of survivin/XIAP mediated aberrant cell survival and increased cell death.

## Discussion

Extensive drug development efforts and clinical evaluations are underway targeting the aberrant cell survival properties associated with PI3K/Akt signaling in regulating cancer progression and metastasis [1]. Inhibition of Akt activation by small molecule kinase inhibitors is an attractive candidate for targeting aberrant cell survival associated with malignant progression and metastasis and could be effective in the treatment of CRC. MK-2206 is a novel Akt allosteric kinase inhibitor, which is currently in clinical evaluation [12-14].

Several studies have described MK-2206 effects as a single agent or in combination with other inhibitors (e.g. PI3K or mTOR inhibitors) on cell proliferation and/or cell death. Gorlick et.al. [32] demonstrated a significant reduction in tumor volume *in vivo* and decreased cell survival *in vitro* in pediatric cancer cell lines following MK-2206 treatment. Simoni et.al. [33] studied the effect of MK-2206 in T cell acute lymphoblastic leukemia demonstrating cell cycle arrest in G0/G1 phase, apoptosis and autophagy. Ma et.al. [34] showed that MK-2206 treatment in nasopharyngeal carcinoma cells (NPC) induced cell cycle arrest and apoptosis. Similarly, we observed that MK-2206 treatment in the IGF1R-dependent GEO cells reduced cell proliferation and increased cell death in a concentration dependent manner (Figure 2A, B) while MK-2206 has been shown to be effective in causing cell death in different types of cancer. However, specific mechanisms associated with MK-2206-mediated cell death have not been characterized. This study identifies molecular mechanisms involved in MK-2206-mediated cell death in IGF1R- dependent CRC cells in response to Akt inhibition. Identification of specific mechanisms may generate new therapeutic targets that offer potential for enhancing cell death of CRC cells. The mechanistic novelty of this study is our identification of 2 pathways whereby MK-2206 treatment leads to control of aberrant cell survival and induction of cell death *in vitro* and *in vivo*.

We studied the expression of various apoptosis-regulators following exposure to MK-2206. As expected, a reduction in phospho-Bad (pBad) at the Ser 136 site was observed (Figure 2D), which is known to be regulated by Akt signaling [29]. It is known that pBad interacts with 14-3-3, a major mediator of cell survival providing an anti-apoptotic milieu to the cellular environment [35]. We observed that treatment with MK-2206 results in reduced 14-3-3 interaction with pBad (Ser136) indicating that MK-2206 results in reduction in cell survival through this mechanism. The protein expression of Bad remained unchanged following MK-2206 treatment; however, there was an increase in the interaction of Bad with Bcl-x_L_. Bad inactivates Bcl-x_L_ thus leading to increases in cell death. Additionally, we observe a decrease in the interaction of Bad with 14-3-3 on treatment with MK-2206. This might suggest that Bad remains activated leading to apoptosis of colorectal cancer cells.

Strikingly, we made the observation that MK-2206 exposure led to an induction of pro-apoptotic protein AIF and its translocation from mitochondria to the nucleus of the GEO cells (Figure 6A, C). It has been reported that AIF is responsible for caspase-independent death in ovarian cancer cells [30,36,37]. AIF is localized in the mitochondria but upon activation it translocates to the nucleus and causes DNA fragmentation [38]. However, the mechanism that regulates AIF induction leading to its caspase-independent apoptotic functions is not well understood. Treatment with AIF inhibitor resulted in reduced cell death thus indicating that AIF is responsible for cell death mediated by MK-2206.

MK-2206 treatment of GEO cells reduced survivin and XIAP levels both *in vivo* and *in vitro* (Figure 2D, Additional file 1: Figure S3). Survivin and XIAP are key cell survival-associated proteins that have been characterized as having an important role in metastasis [39]. XIAP binds to caspases 3, 7 and 9 thereby inhibiting their pro-apoptotic activity [39,40]. During stress conditions, mitochondrial XIAP and survivin migrate to the cytosol forming a survivin/XIAP complex, which inhibits caspases and promotes cytoprotection [40]. Dan et al. [41] made the novel finding that Akt phosphorylates XIAP at a stabilizing Ser87 site. We demonstrated that TGFβ/PKA signaling regulates aberrant cell survival in IGF1R-dependent CRC cells by disengaging survivin/XIAP complex formation thus causing caspase activation and inducing cell death. We sought to determine the mechanism by which MK-2206 increased XIAP loss and cell death. It was observed that MK-2206 treatment dephosphorylates Ezrin at the Thr567 site (Figure 7A, B). However, no change in total Ezrin protein expression was observed. Ezrin is a member of Ezrin-radixin-moesin (ERM) protein family that plays a key role in cancer progression and metastasis in a wide range of cancers, including CRC [42]. Ezrin is found in a closed confirmation in the cytosol. Ezrin phosphorylation at Thr567 leads to its activation and conformational change to an open conformation resulting in its localization to the plasma membrane for its oncogenic-associated functions [43]. Several kinases are known to phosphorylate Ezrin at T567 including Rho kinase and PI3K/Akt [44]. We performed siRNA knockdown of Ezrin and observed a complete loss of XIAP and survivin (Additional file 1: Figure S11). Thus, we have found that MK-2206 treatment inhibits the Akt-pEzrinT567-XIAP cell survival-signaling axis leading to a caspase-dependent cell death in the IGF1R-dependent CRC cells, in addition to caspase independent cell death accompanying AIF translocation from the mitochondria to the nucleus.

Stable knockdown of Akt2 in the IGF1R-dependent and highly metastatic colon cancer cell line GEO was performed to give a better understanding of the mechanism of cell death mediated by loss of pEzrin. Loss of Akt2 resulted in decreased the activation of Ezrin since there was a loss of phosphorylation of Ezrin at the T567 site. Besides loss of pEzrin we also observed a reduction in the expression of XIAP on knockdown of Akt2. However, there was no such loss of pEzrin on knockdown of Akt1 and Akt3 in GEO cells. Thus we can conclude that loss of the Akt2 isoform is responsible for Akt-pEzrin-XIAP mediated cell death.

## Conclusion

We provided novel mechanistic insights on MK-2206-mediated cell death. Importantly, this work provides a new paradigm for MK-2206-mediated control of aberrant cell survival associated with IGF1R-dependent CRC that may offer new targets for enhancing cell death in cancer cells.

## Abbreviations

CRC: Colorectal cancer; IGF1R: Insulin-like growth factor receptor 1; PI3K: Phosphoinositide 3-kinase; AIF: Apoptosis inducing factor.

## Competing interests

The authors declare that they have no competing interests.

## Authors’ contributions

EA carried out the majority of the in vitro and in vivo experiments in the study analyzed the data and drafted the manuscript. ABC and PDL helped with the in vivo studies. KLH helped with performing western blots. MGB and SC participated in the conception and design of the study and helped to draft the final manuscript. All authors read and approved the final manuscript.

## Pre-publication history

The pre-publication history for this paper can be accessed here:

http://www.biomedcentral.com/1471-2407/14/145/prepub

## Supplementary Material

Additional file 1: Figure S1 Western blot analysis showing a loss of pAkt (S473) after treatment with MK-2206 in HCT116 and MiaPaCa cells. **Figure S2.** Transfection with siRNA for XIAP results in increase in cell death as determined by DNA fragmentation. **Figure S3.** Western blot analysis to determine the loss of survivin and XIAP in animals treated with MK-2206. **Figure S4.** There was no significant loss of body weight in mice on treatment with MK-2206. **Figure S5.** IHC images showing no change in the expression of total Akt in treated animals as compared to control. **Figure S6.** Relative quantification followed by statistical analysis was performed to determine the change in expression of total Akt. There was no significant change in the expression of total Akt. **Figure S7.** Eosin and Hematoxylin staining of control and treated xenograft tumors. **Figure S8.** Images of control and treated animals before euthanizing. **Figure S9.** A) Western blot showing a knockdown of AIF in presence of siRNA. B) DNA fragmentation after knockdown of AIF shows reduction in cell death in presence and absence of MK-2206. **Figure S10.** No change in pEzrin (T567) and total Ezrin on knockdown of Akt3. **Figure S11.** siRNA-mediated knockdown of Ezrin showing a loss in XIAP expression. Click here for file
