# Supplementary material for: Akt inhibitor MK-2206 promotes anti-tumor activity and cell death by modulation of AIF and Ezrin in colorectal cancer
Source: BMC Cancer. 2014 Mar 1;14:145. doi: 10.1186/1471-2407-14-145 (PMC3941258; doi:10.1186/1471-2407-14-145)
Supplement: Additional file 1: Figure S1 — Western blot analysis showing a loss of pAkt (S473) after treatment with MK-2206 in HCT116 and MiaPaCa cells. Figure S2. Transfection with siRNA for XIAP results in increase in cell death as determined by DNA fragmentation. Figure S3. Western blot analysis to determine the loss of survivin and XIAP in animals treated with MK-2206. Figure S4. There was no significant loss of body weight in mice on treatment with MK-2206. Figure S5. IHC images showing no change in the expression of total Akt in treated animals as compared to control. Figure S6. Relative quantification followed by statistical analysis was performed to determine the change in expression of total Akt. There was no significant change in the expression of total Akt. Figure S7. Eosin and Hematoxylin staining of control and treated xenograft tumors. Figure S8. Images of control and treated animals before euthanizing. Figure S9. A) Western blot showing a knockdown of AIF in presence of siRNA. B) DNA fragmentation after knockdown of AIF shows reduction in cell death in presence and absence of MK-2206. Figure S10. No change in pEzrin (T567) and total Ezrin on knockdown of Akt3. Figure S11. siRNA-mediated knockdown of Ezrin showing a loss in XIAP expression. [file 1471-2407-14-145-S1.pptx]

## Slide 1
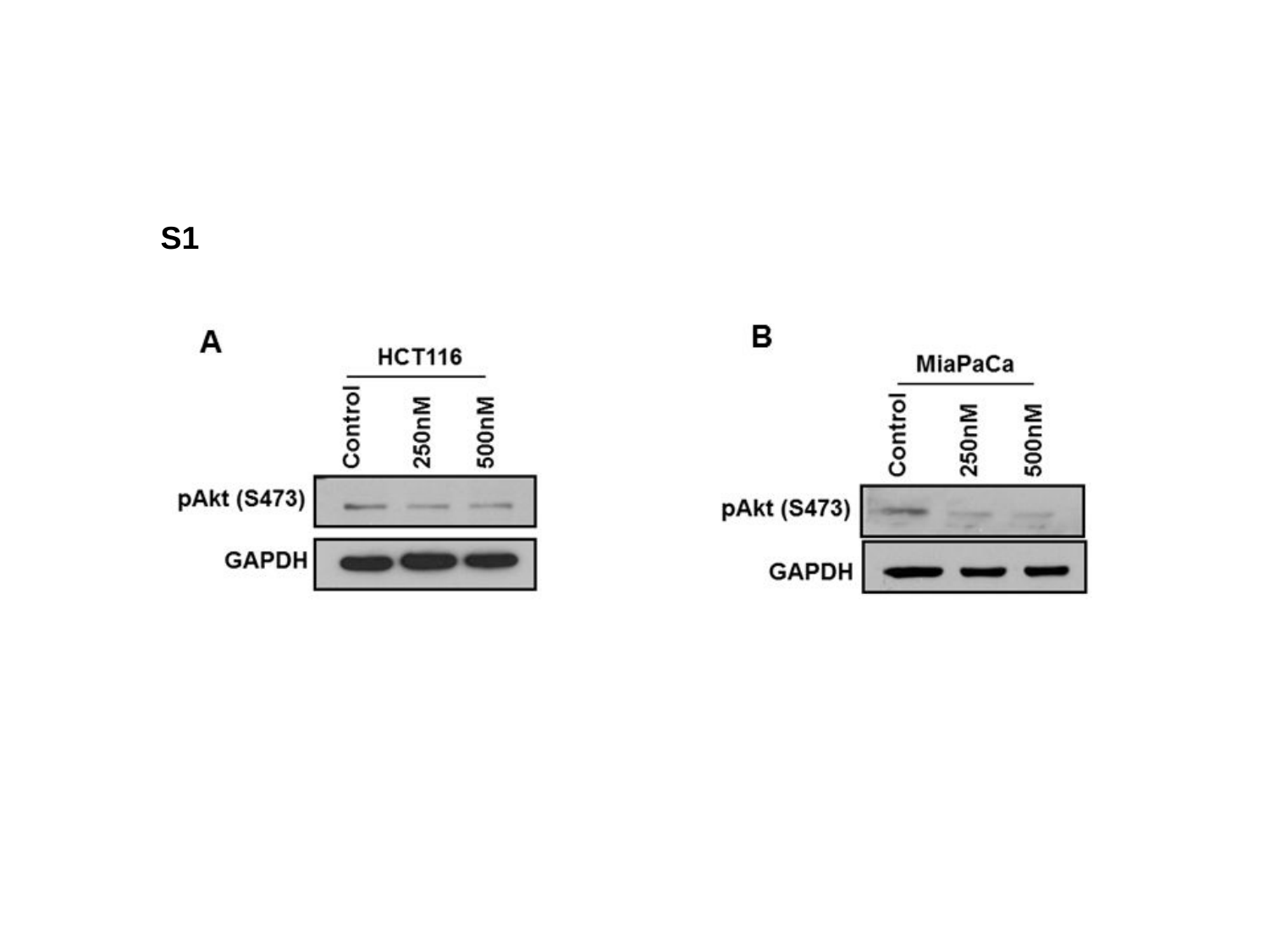

S1

## Slide 2
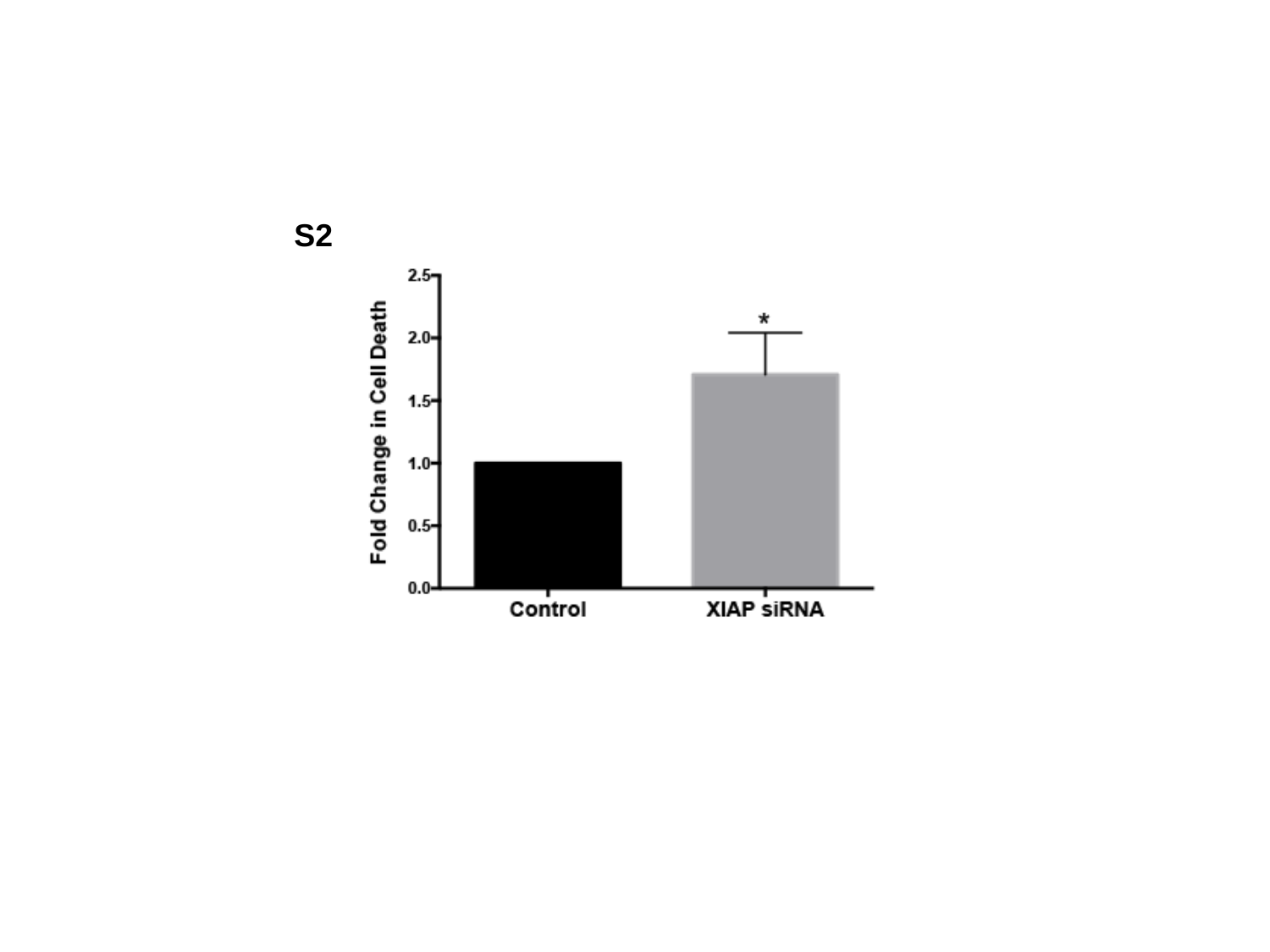

S2

## Slide 3
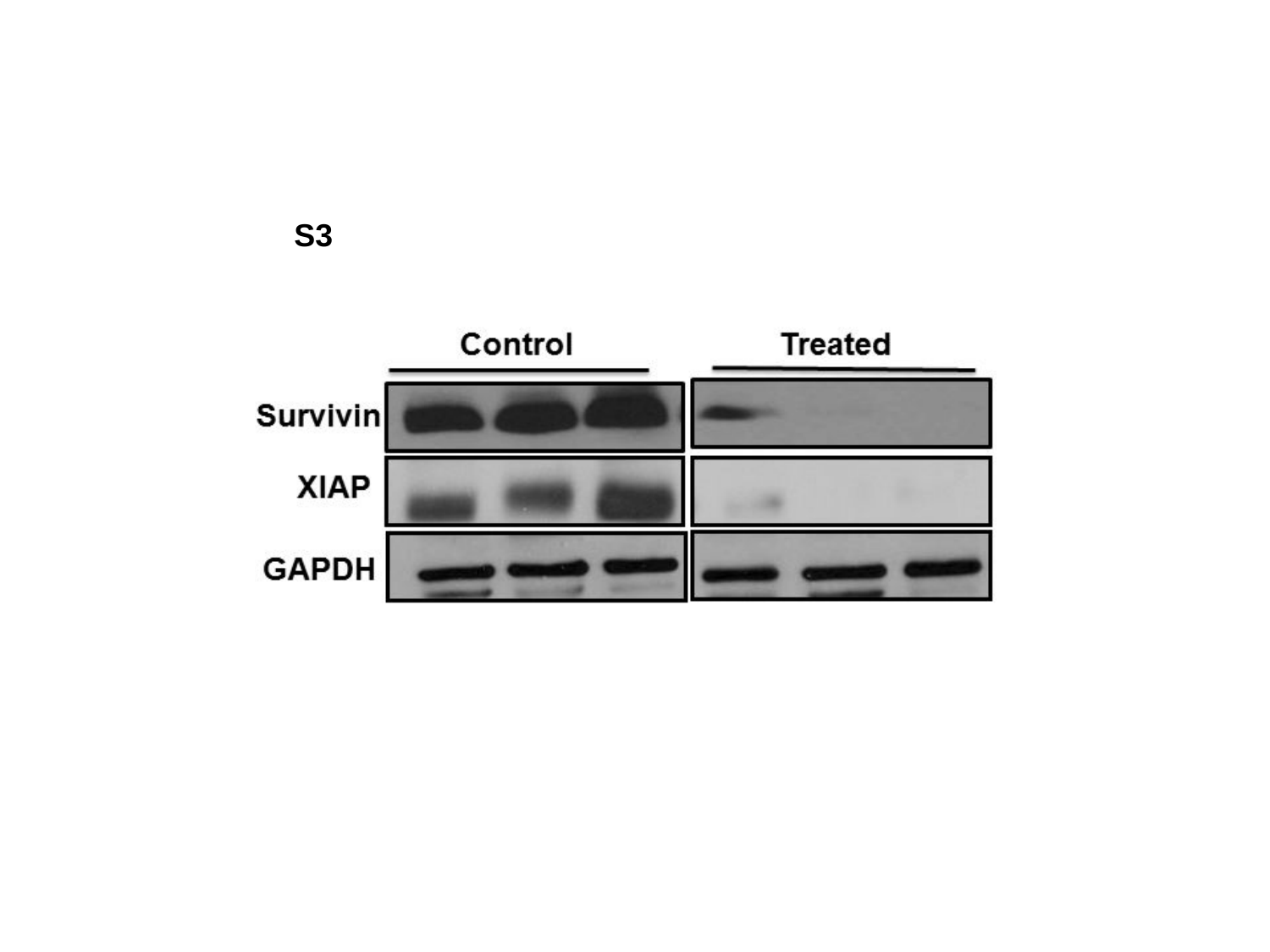

S3

## Slide 4
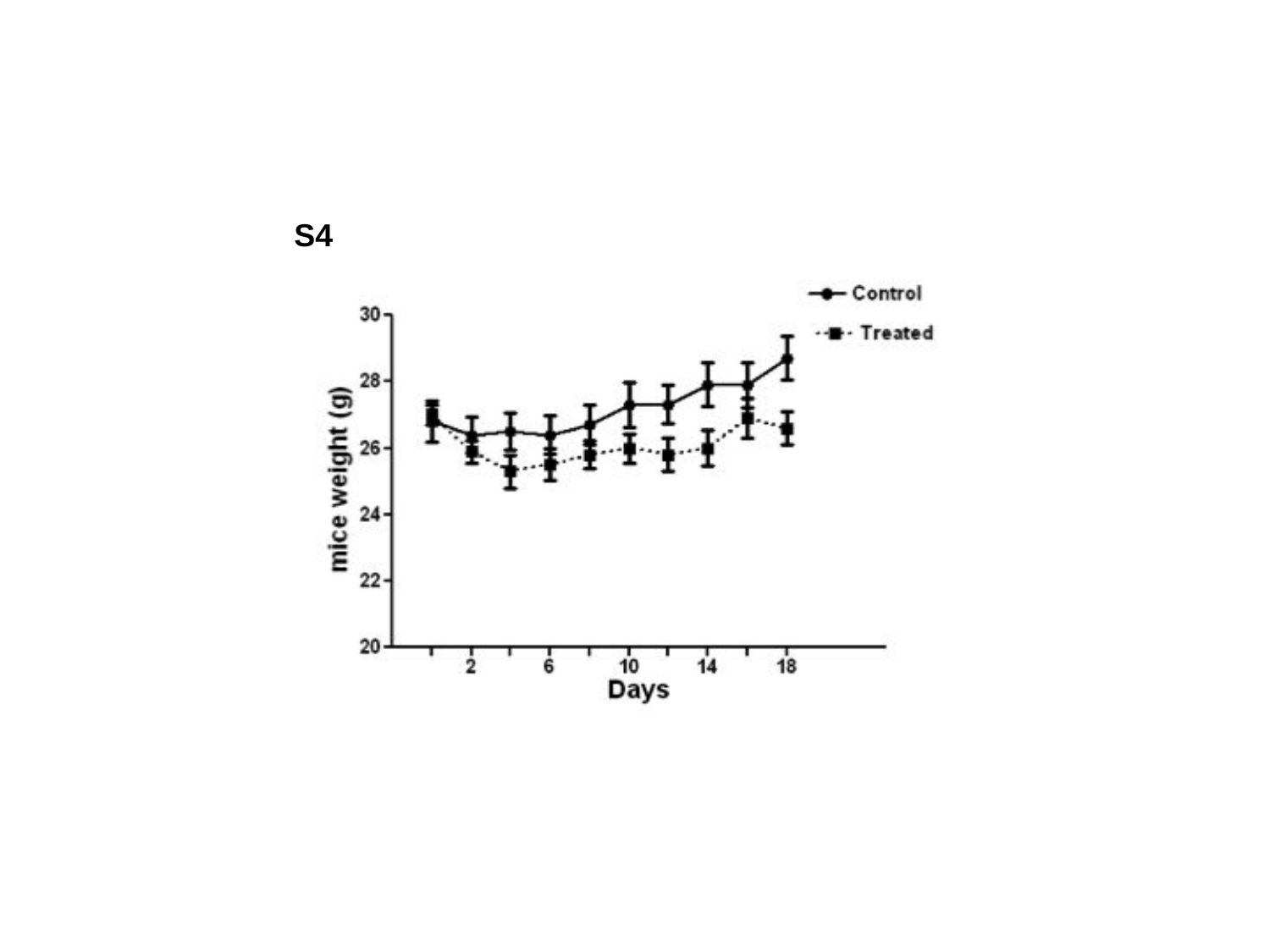

S4

## Slide 5
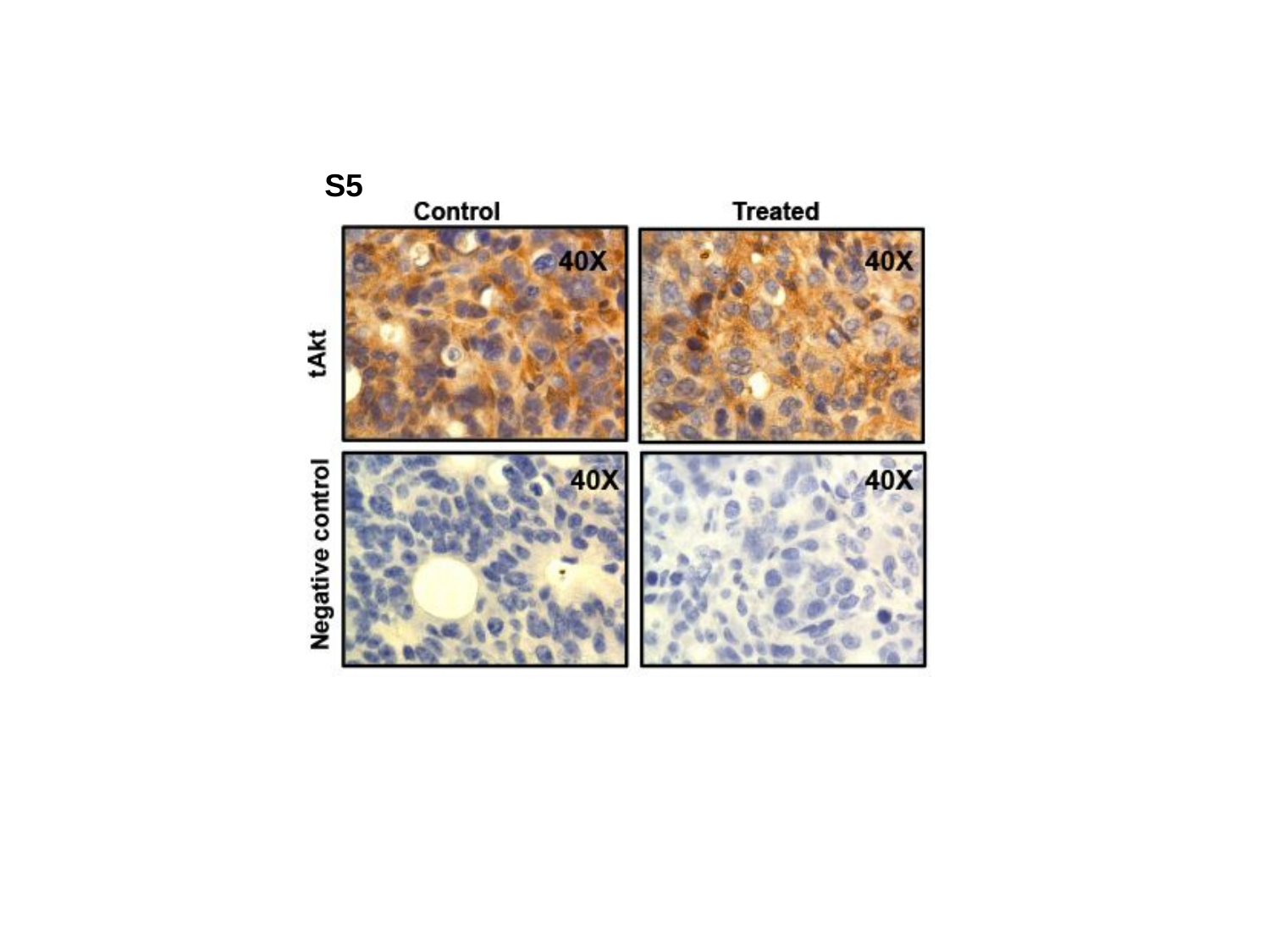

S5

## Slide 6
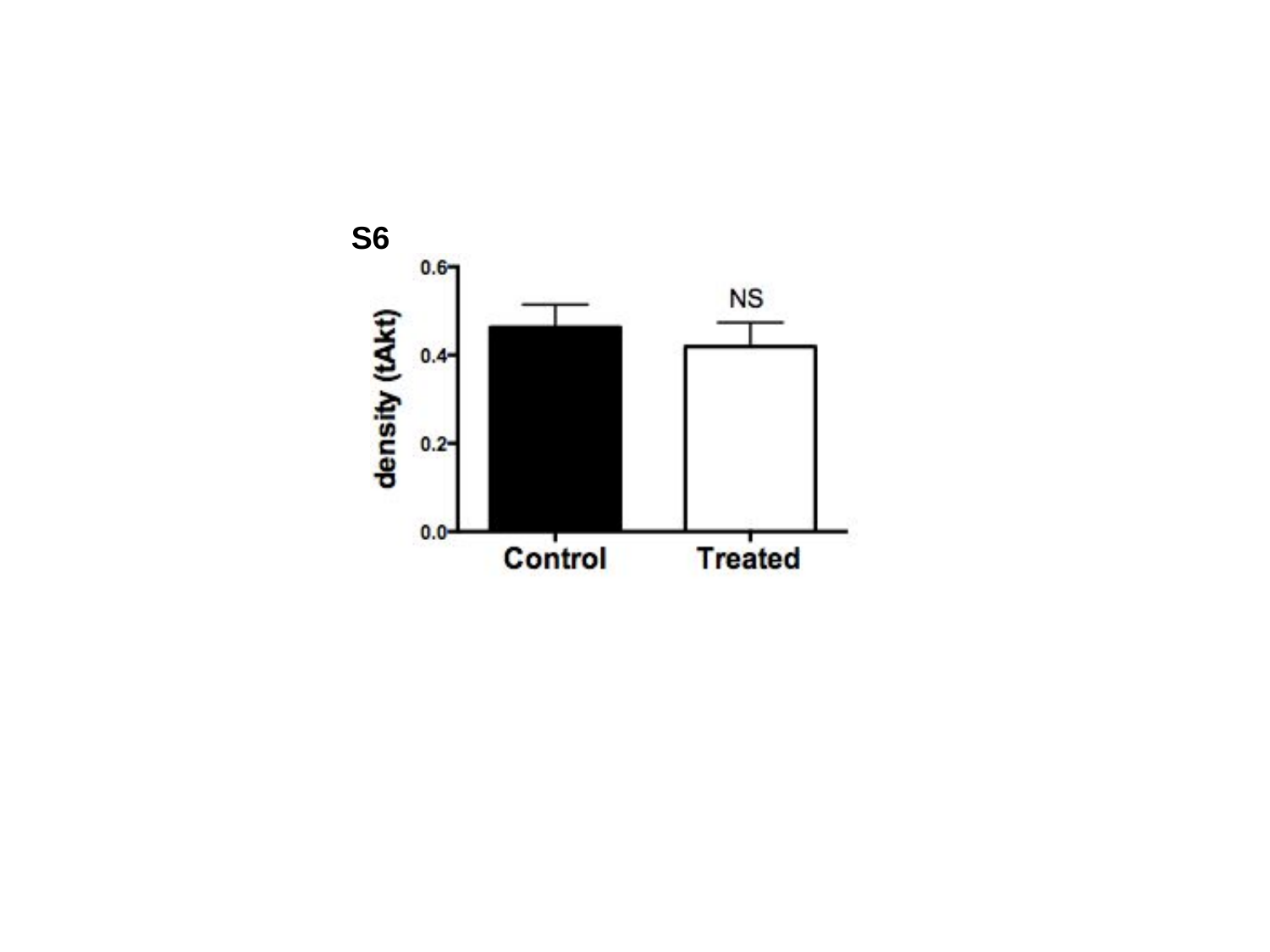

S6

## Slide 7
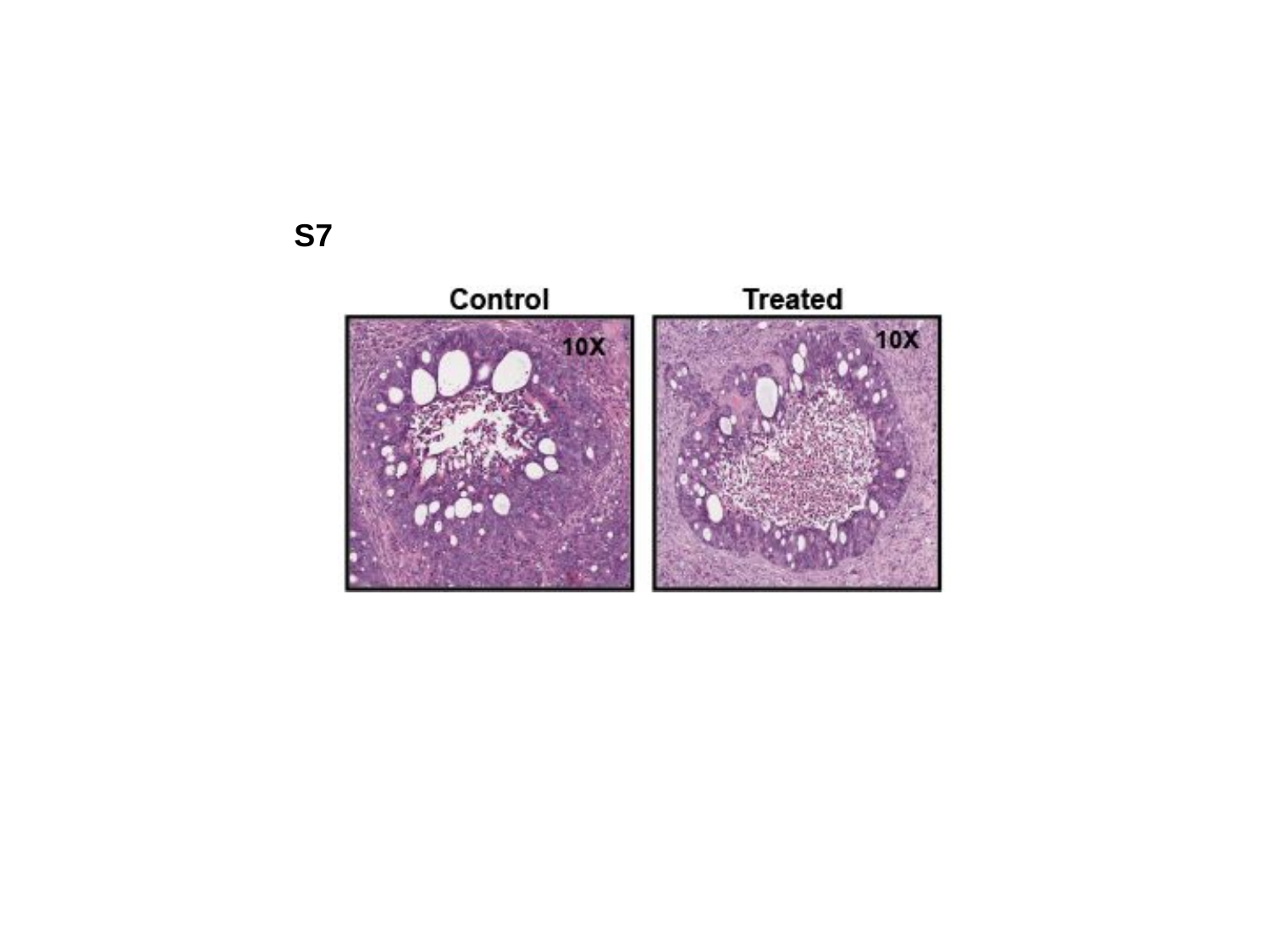

S7

## Slide 8
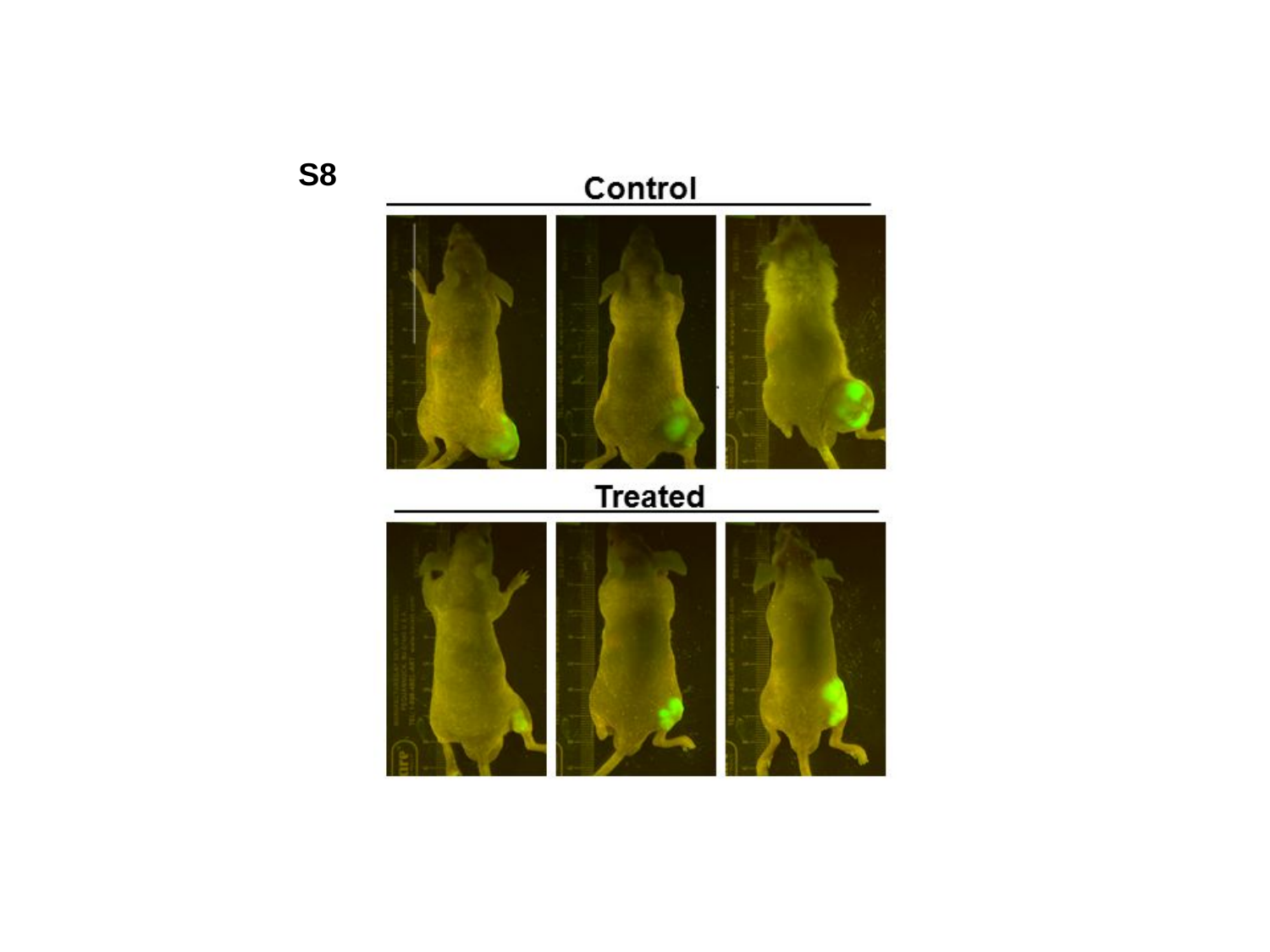

S8

## Slide 9
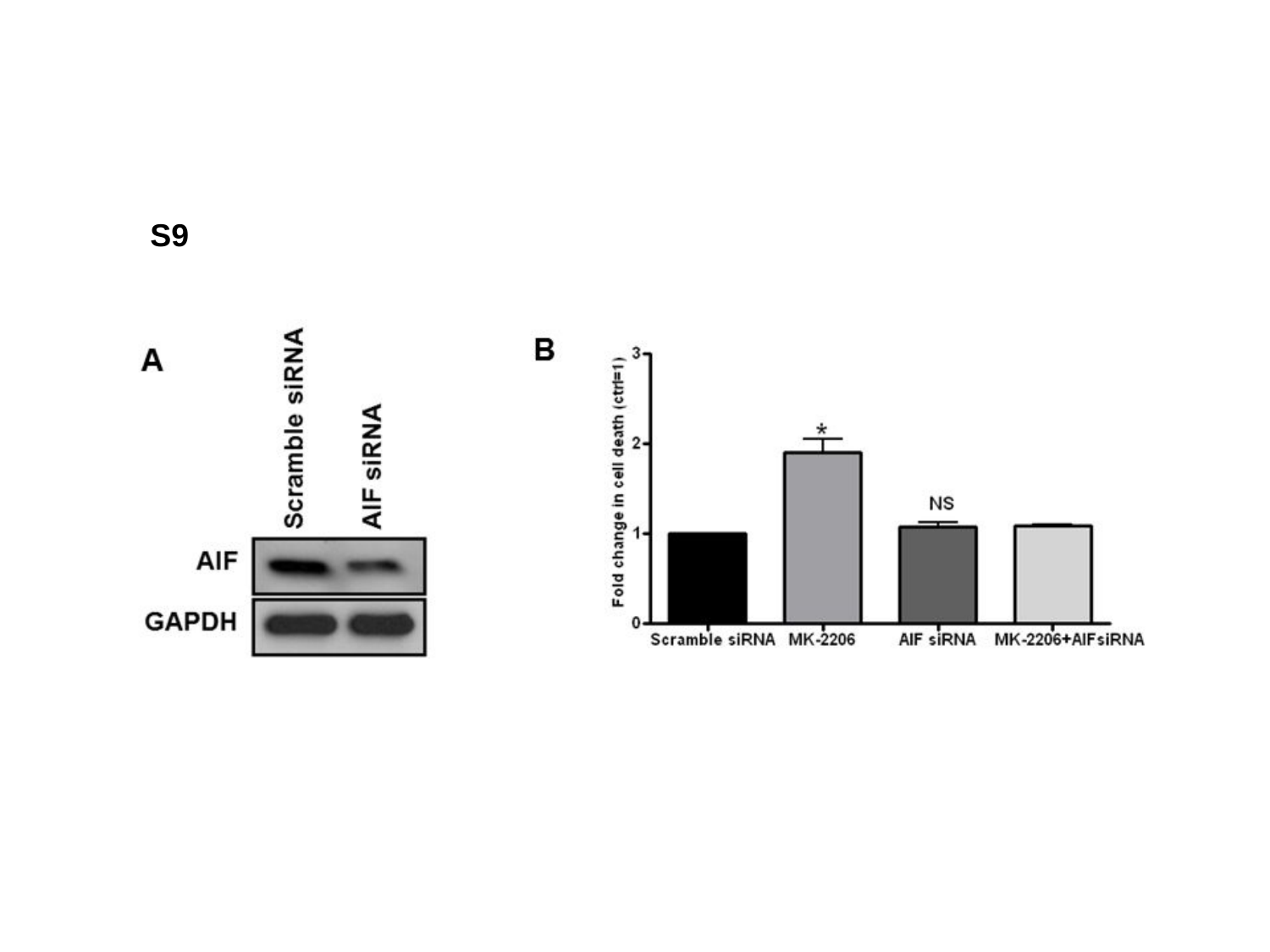

S9

## Slide 10
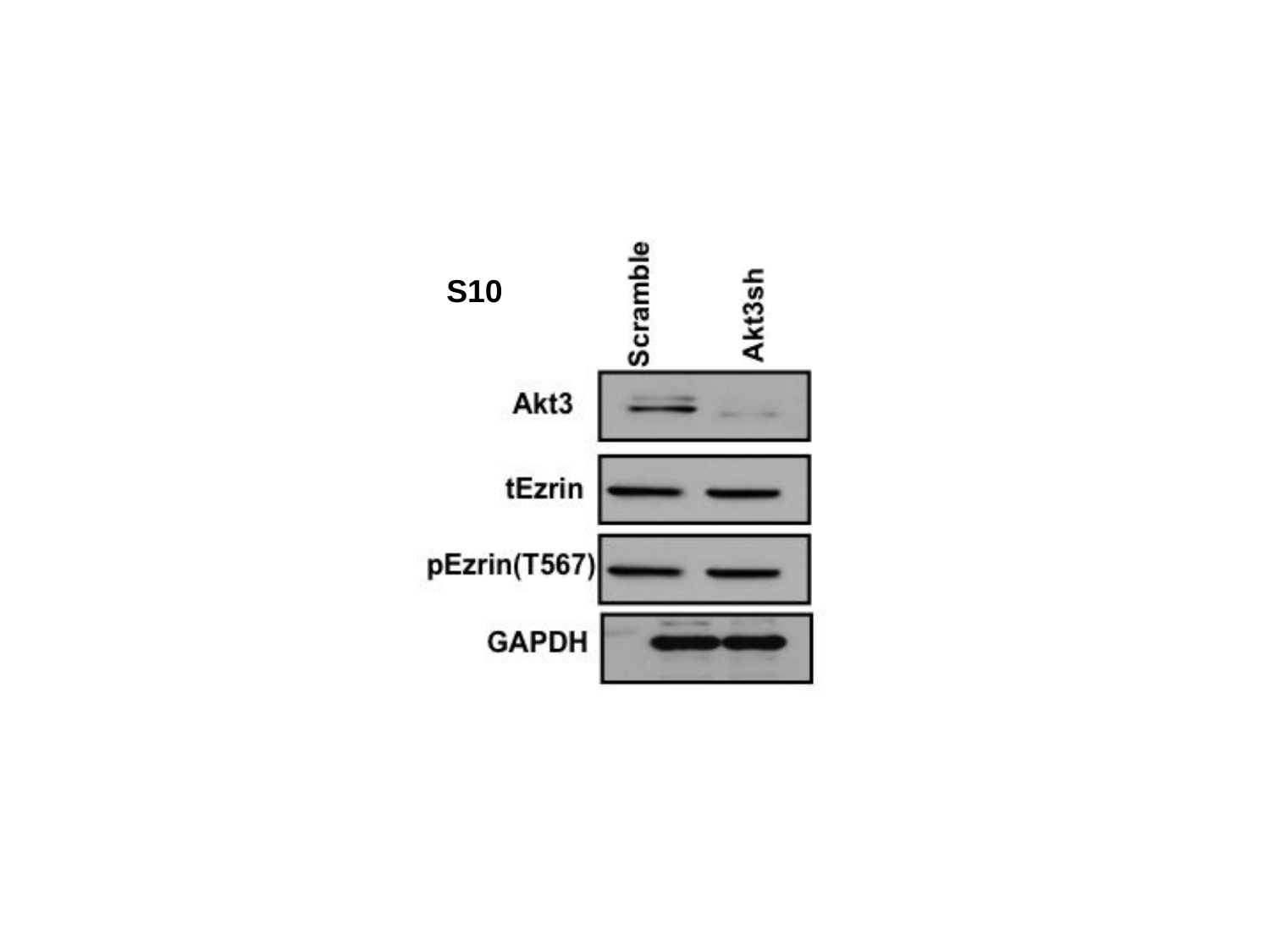

S10

## Slide 11
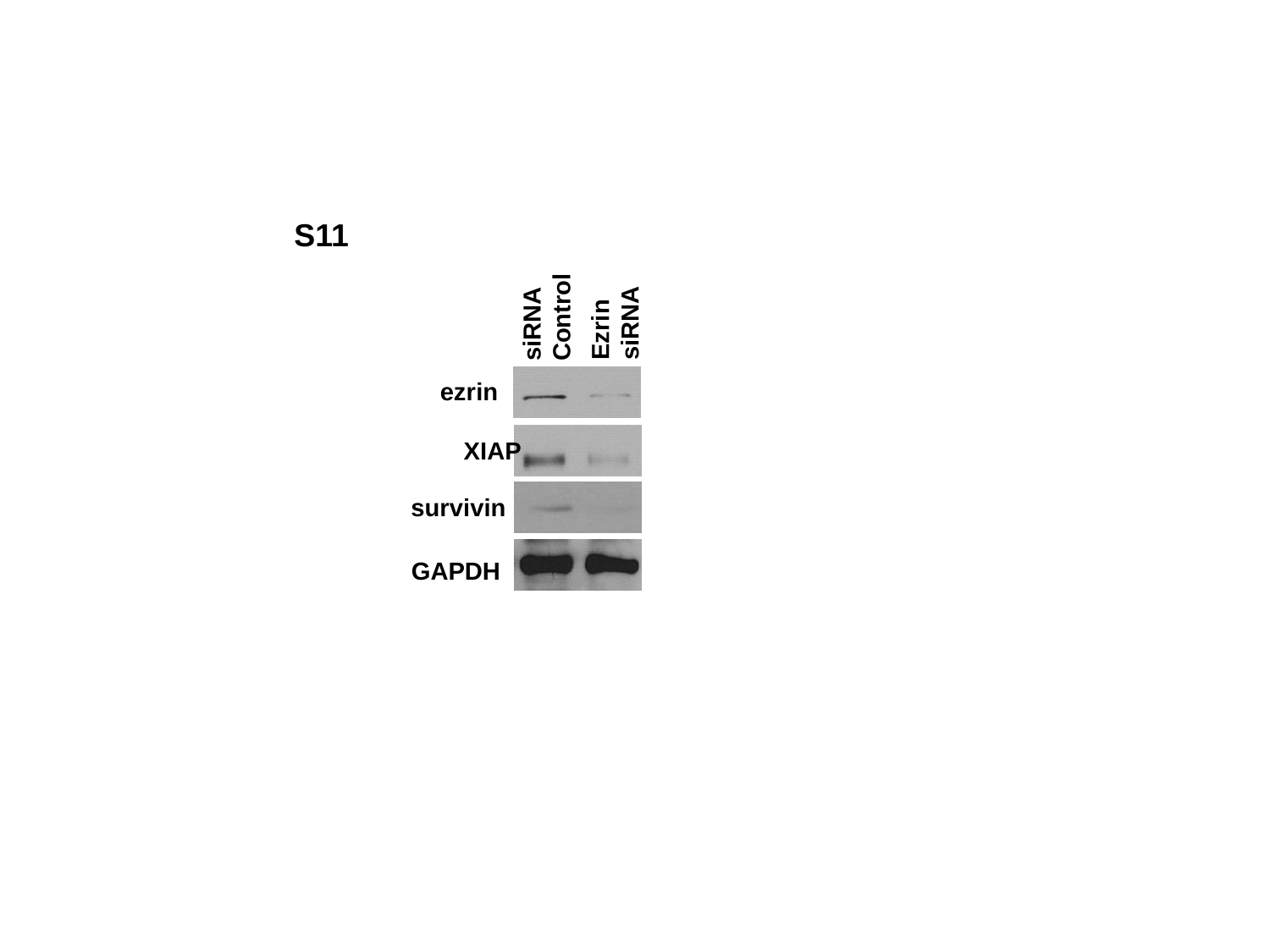

siRNA
Control
Ezrin
siRNA
ezrin
XIAP
survivin
GAPDH
S11
